# Supplementary material for: Towards unified AI-driven fracture mechanics: the extended deep energy method (XDEM)
Source: Nat Commun. 2026 Aug 15;17:8492. doi: 10.1038/s41467-026-76748-1 (PMC13484471; doi:10.1038/s41467-026-76748-1)
Supplement: Supplementary file 3 [file 41467_2026_76748_MOESM3_ESM.zip › pic/discussion/loss_landscape/loss_DEM_XDEM.pdf]

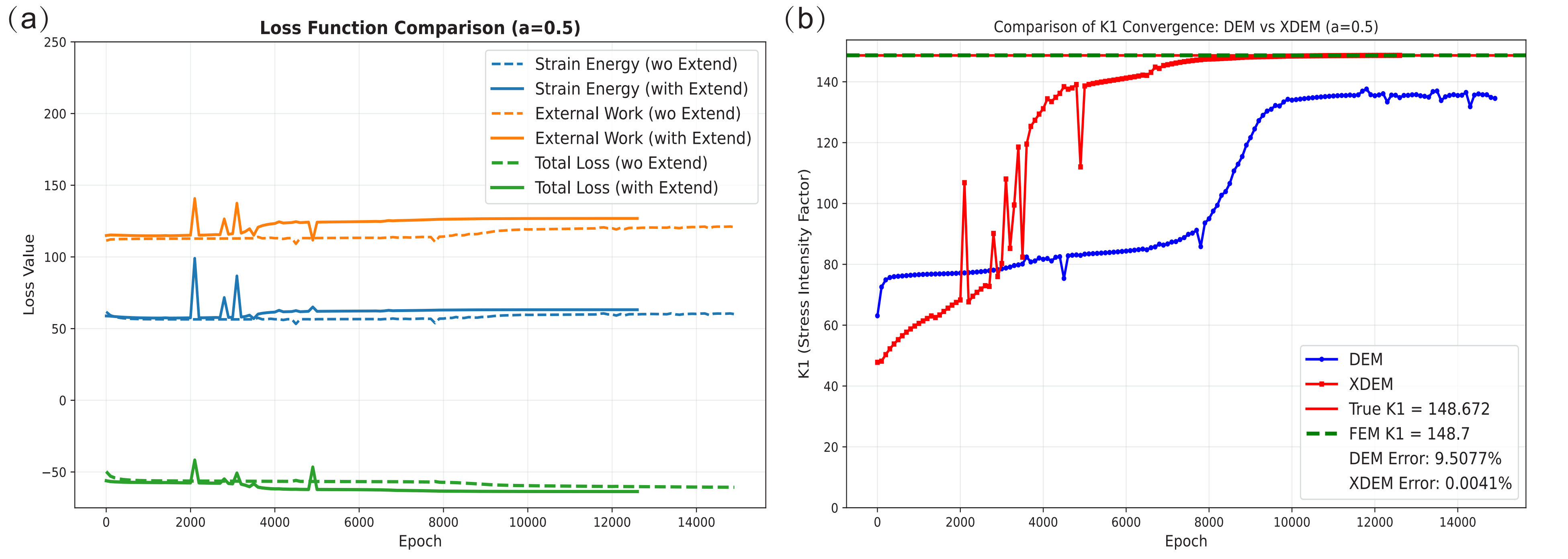
(a)
Loss Function Comparison (a=0.5)
Strain Energy (wo Extend)
Strain Energy (with Extend)
External Work (wo Extend)
External Work (with Extend)
Total Loss (wo Extend)
Total Loss (with Extend)
Loss Value
Epoch
(b)
Comparison of K1 Convergence: DEM vs XDEM (a=0.5)
DEM
XDEM
True K1 = 148.672
FEM K1 = 148.7
DEM Error: 9.5077%
XDEM Error: 0.0041%
K1 (Stress Intensity Factor)
Epoch
